# Sleep quality and psychological health in patients with pelvic and acetabulum fractures: a cross-sectional study

**DOI:** 10.1186/s12877-024-04929-y

**Published:** 2024-04-04

**Authors:** Khan Akhtar Ali, LingXiao He, Wenkai Li, Weikai Zhang, Hui Huang

**Affiliations:** Department of Orthopedics, Tongji Hospital, Tongji Medical College, Huazhong University of Science and Technology, Jiefang Avenue, Qiaokou District, Wuhan, Hubei 430030 China

**Keywords:** Acetabulum, Pelvic, Fractures, Sleep disorders, Anxiety, Depression, Functional outcome

## Abstract

**Background and objectives:**

It is known that difficulty sleeping after a fracture can have negative effects on both mental and physical health and may prolong the recovery process. The objective of this study is to explore how sleep quality and psychological health are linked in patients with pelvic and acetabulum fractures.

**Methods:**

A study was conducted on 265 patients between 2018 and 2022 who had suffered pelvic and acetabulum fractures. The study examined various factors, including age, gender, cause of injury, post-operative complications, and injury severity. The study employed ordinal logistic regression to examine the relationship between various pelvic fractures and seven subscales of the Majeed Pelvic Score (MPS), as well as the Sleep Disorder Questionnaire (SDQ) and Beck Depression Inventory (BDI). The study focused on the postoperative outcome one year after surgery, and each patient was assessed at the one-year mark after surgical intervention. Additionally, the study evaluated the functional outcome, sleep quality, and psychological disorders of the patients.

**Results:**

From 2018 to 2022, a total of 216 patients suffered from pelvic and acetabulum fractures. Among them, 6.6% experienced borderline clinical depression, and 45.2% reported mild mood disturbances. Anxiety was found to be mild to moderate in 46% of Tile C and posterior acetabulum wall fracture patients. About 24.8% of patients reported insomnia, while 23.1% reported sleep movement disorders. However, no significant correlation was found between fracture types and sleep disorders. The mean Majeed pelvic score (MPS) was 89.68.

**Conclusions:**

Patients with pelvic and acetabular fractures typically experience functional improvement, but may also be at increased risk for insomnia and sleep movement disorders, particularly for certain types of fractures. Psychological well-being varies between fracture groups, with signs of borderline clinical depression observed in some cases. However, anxiety levels do not appear to be significantly correlated with pelvic and acetabular fractures.

**Supplementary Information:**

The online version contains supplementary material available at 10.1186/s12877-024-04929-y.

## Introduction

The term “pelvic fracture” refers to both acetabular and pelvic ring fractures originating from high- and low-energy trauma. In young patients, these fractures typically result from high-energy trauma, but in elderly patients, they more frequently result from low-energy trauma [1, 2]. Pelvic and acetabular trauma frequently manifests as polytrauma and may result in fatal hemodynamic instability [3]. After a pelvis fracture, the functional result and health-related quality of life (HRQOL) are not excellent; most patients do not resume their previous activities [4]. After an acute orthopedic injury, nearly one-third of patients experience depression, and more than one-quarter experience PTSD [5]. After pelvic ring fractures, chronic posttraumatic pelvic pain hurts concerns with quality of life [6]. Patients with orthopedic trauma injuries are significantly more likely to experience psychological distress [7]. In addition to these physiologic studies, posttraumatic psychological issues like posttraumatic stress disorder, chronic pain, depression, and/or anxiety seem to play a role in the negative functional outcomes [7–9]. Patients with depression and those with chronic pelvic pain have various sleep patterns [10]. A significant number of patients with post-traumatic stress disorder (PTSD)following injury also have depression, according to studies. One study found that four months after injury, 16 of 37 PTSD patients also had major depression. Patients who have both major depression and PTSD simultaneously seem to experience more problems than those who only have one of the two conditions. In comparison to people who only have PTSD or major depression, Shalev et al. discovered that patients with comorbid PTSD and depression reported more symptoms, felt more distress from those symptoms, and performed worse in daily life [11, 12]. Orthopedic trauma patients often suffer from anxiety and depression, which can lead to negative surgical outcomes. Psychological distress, chronic pain, and traumatic limb amputation also contribute to adverse mental health outcomes [13].Depression is a widespread mental health disorder that affects a significant portion of the world’s population, leading to a heavy burden on society [14–16]. Following orthopedic trauma, the patient psychological status has received less attention [17]. Sleep is crucial for rest, recovery, information processing, and memory consolidation. After surgery, sleep deprivation can cause changes in the sleep cycle, such as the absence of rapid eye movement (REM) due to pain caused by inflammation. Surgical trauma may also lead to immunosuppression, which increases the risk of infection [18–21]. High levels of the cytokine IL-6 at night can cause disrupted and superficial sleep, while low levels are linked to deep and restful sleep. Surgical inflammation may also contribute to postoperative sleep disturbances, with major surgeries leading to the most significant disruption [22–24]This cross-sectional study aims to investigate how sleep quality and psychological health affect patients with pelvic and acetabulum fractures. The study evaluates the functional outcome, sleep quality, and psychological disorders of the patients one year after surgical intervention. The study also examines the relationship between various pelvic fractures and seven subscales of the Majeed Pelvic Score (MPS), as well as the Sleep Disorder Questionnaire (SDQ) and Beck Depression Inventory (BDI). The hypothesis is that patients with pelvic and acetabular fractures typically experience functional improvement but may also be at an increased risk for insomnia and sleep movement disorders, particularly for certain types of fractures. psych logicalizes that psychological well-being varies between fracture groups, with signs of borderline clinical depression observed in some cases.

## Patients and methods

The hospital database initially had 265 people, but due to some unfortunate circumstances, contact information for 48 patients was lost or modified, and one patient passed away. This left us with only 216 patients, or 82% of the initial group, who were eligible for the study. We contacted these patients and asked them some questions before starting the interview using related questionnaires. To ensure a controlled study, we confirmed whether the patients were currently taking any anti-psychotic drugs or NSAIDs. We also asked if they had any psychological or sleep disorders problems prior to their pelvic fractures. After selecting the patients who met the criteria, we interviewed them using questionnaires to assess sleep problems, anxiety, and depression. 82% of patients completed the questionnaires with no significant difference in age, gender, Tile categorization or injury severity score (ISS) compared to those not contacted. The data of the remaining 18% were analyzed retrospectively due to lost or changed contact information, including age, gender, tile categorization, and ISS. The study evaluated various factors, including age, gender, injury severity, and post-operative complications, and assessed the functional outcome, sleep quality, and psychological disorders of the patients. The study aimed to clarify the impact of sleep and psychiatric disorders on patients with pelvic and acetabular fractures. To evaluate the functional outcome of pelvic fractures and acetabulum and to correlate them with sleep and psychological disorders, we used the Majeed pelvic score. To minimize the negative impact of concurrent injuries on the health of patients, we assessed them one year after surgical intervention for pelvic and acetabulum fractures. To ensure accuracy, patients with severe and multiple injuries related to pelvic trauma were excluded from the study based on our criteria. The Majeed Pelvic Score (MPS) is a widely used health-related quality of life instrument that assesses pain, work, sitting, sexual intercourse, standing, walking aids, unaided gait, and walking distance. The MPS score ranges from 16 (worst health state) to 100 (best health state) [25–27]. Baker et al. published the Injury Severity Score (ISS) in 1974, which describes the severity and death probability in patients with multiple injuries. The ISS compares injuries and outcomes retrospectively and is easily accessible to clinicians and researchers. The ISS has been the standard for trauma scoring for over 20 years [28]. Tile classification: Tile developed a classification system for pelvis bone fractures based on the observed injury mechanisms, which include anterior-posterior compression, lateral compression, and vertical shear. His classification shows that the mortality rates increase from type A to type C fractures, with the highest mortality rates occurring after C2 injuries. Additionally, B3 fractures have comparable mortality rates to C-type fractures [29]. The Judet and Letournel classification system is widely used by orthopedic surgeons to determine the appropriate surgical approach for acetabular fractures. Certain fracture patterns in the classification have worse prognostic outcomes [30].

### Inclusion exclusion criteria

**Fig. 1:**
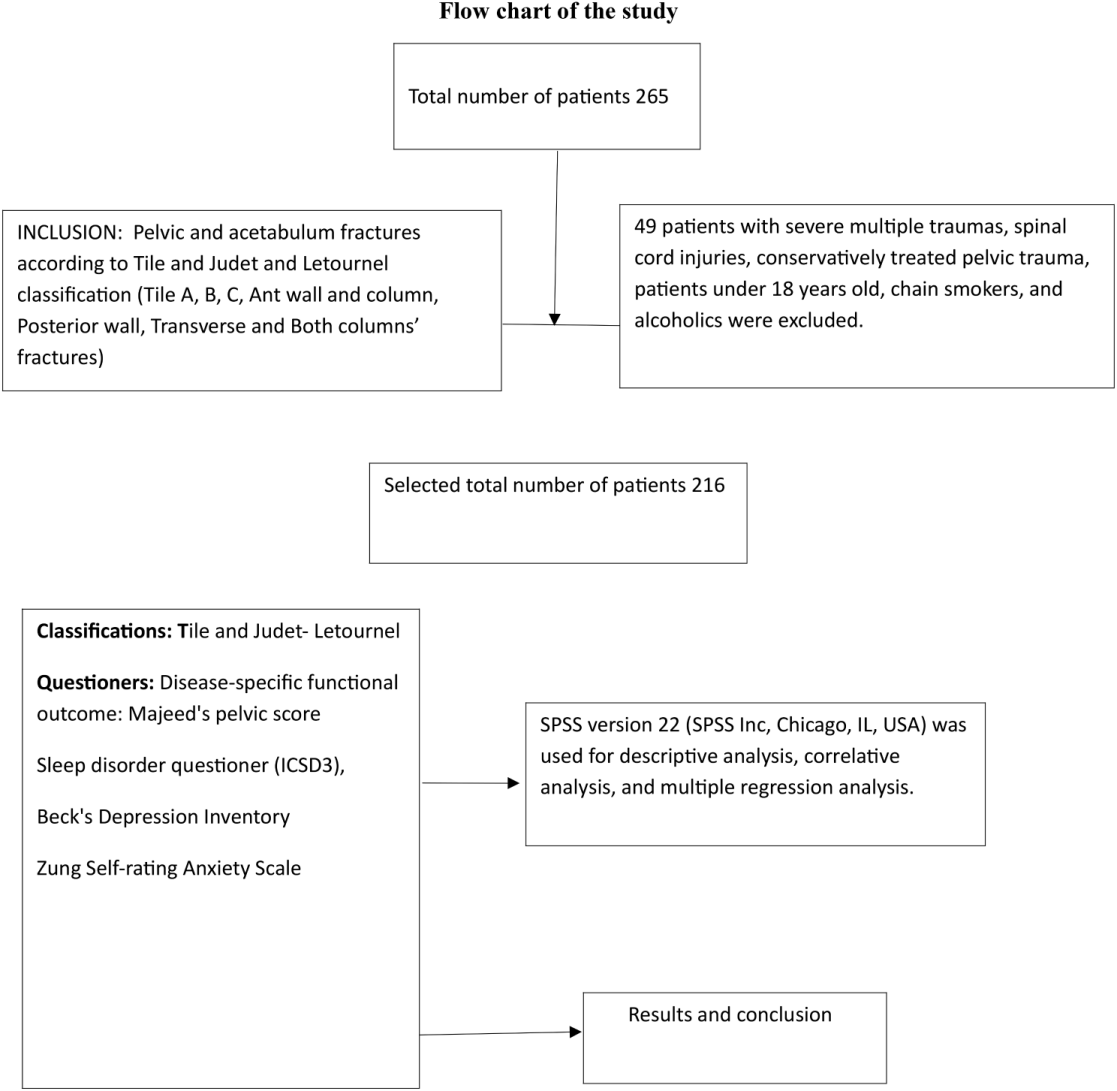
Study flow chart

The study included cases of pelvic and acetabulum fractures classified according to Tile and Judet and Letournel classification, which included Tile A, B, C, Ant wall and column, Posterior wall, Transverse and Both columns’ fractures. However, the study excluded patients with multiple severe trauma, spinal cord injuries, patients who were conservatively treated for pelvic trauma, patients under the age of 18, chain smokers and alcoholics. Additionally, patients who were on anti-psychotic or anti-epileptic drugs, had neurological disorders, were using Parkinson or Anti-Parkinsonian drugs, and those on sleeping pills and antidepressive drugs were also excluded from this study.(Fig. 1).

### Sleeping disorders questionnaire

**Fig. 2:**
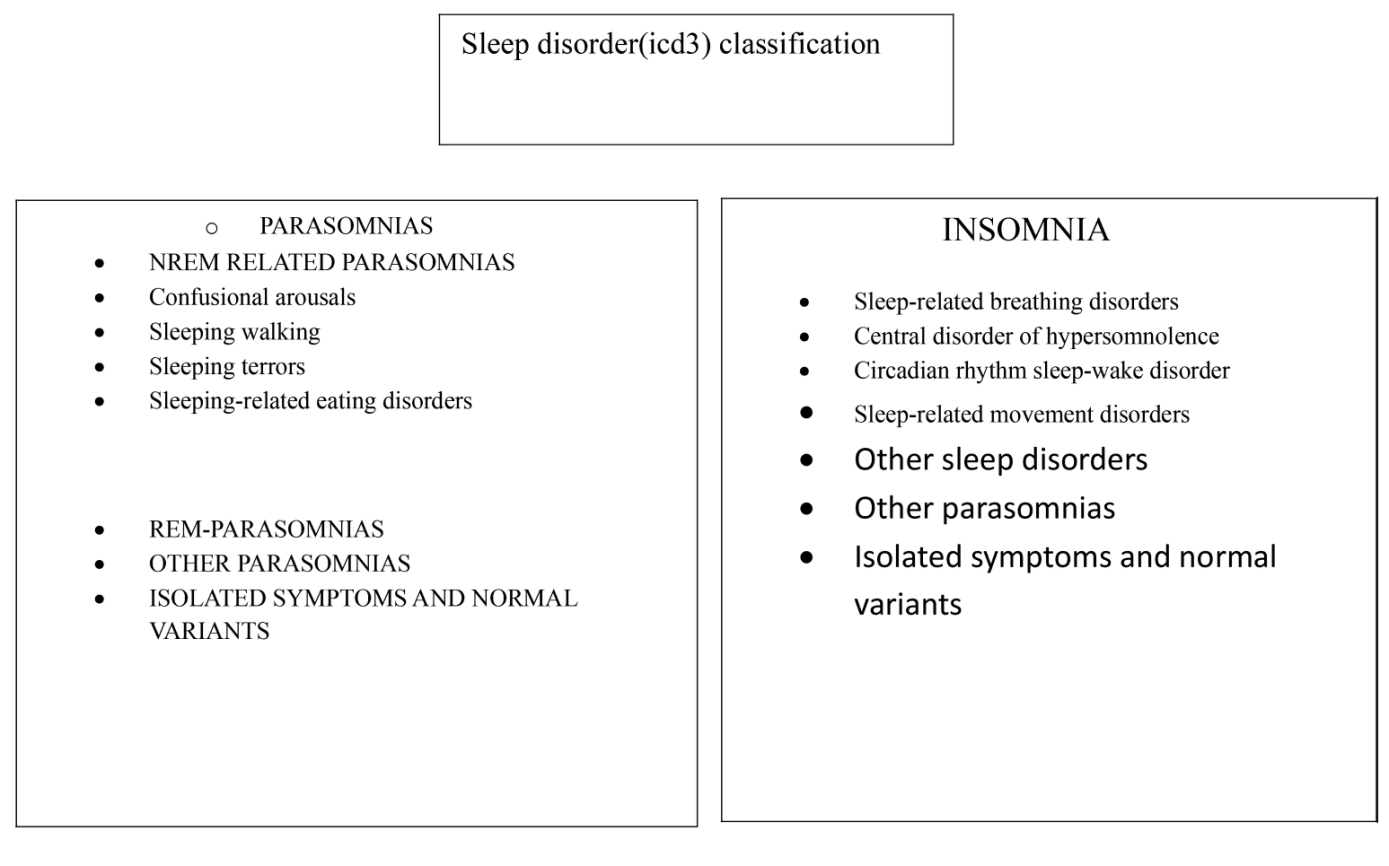
International classification of sleep disorders (ICSD3)

**Fig. 3:**
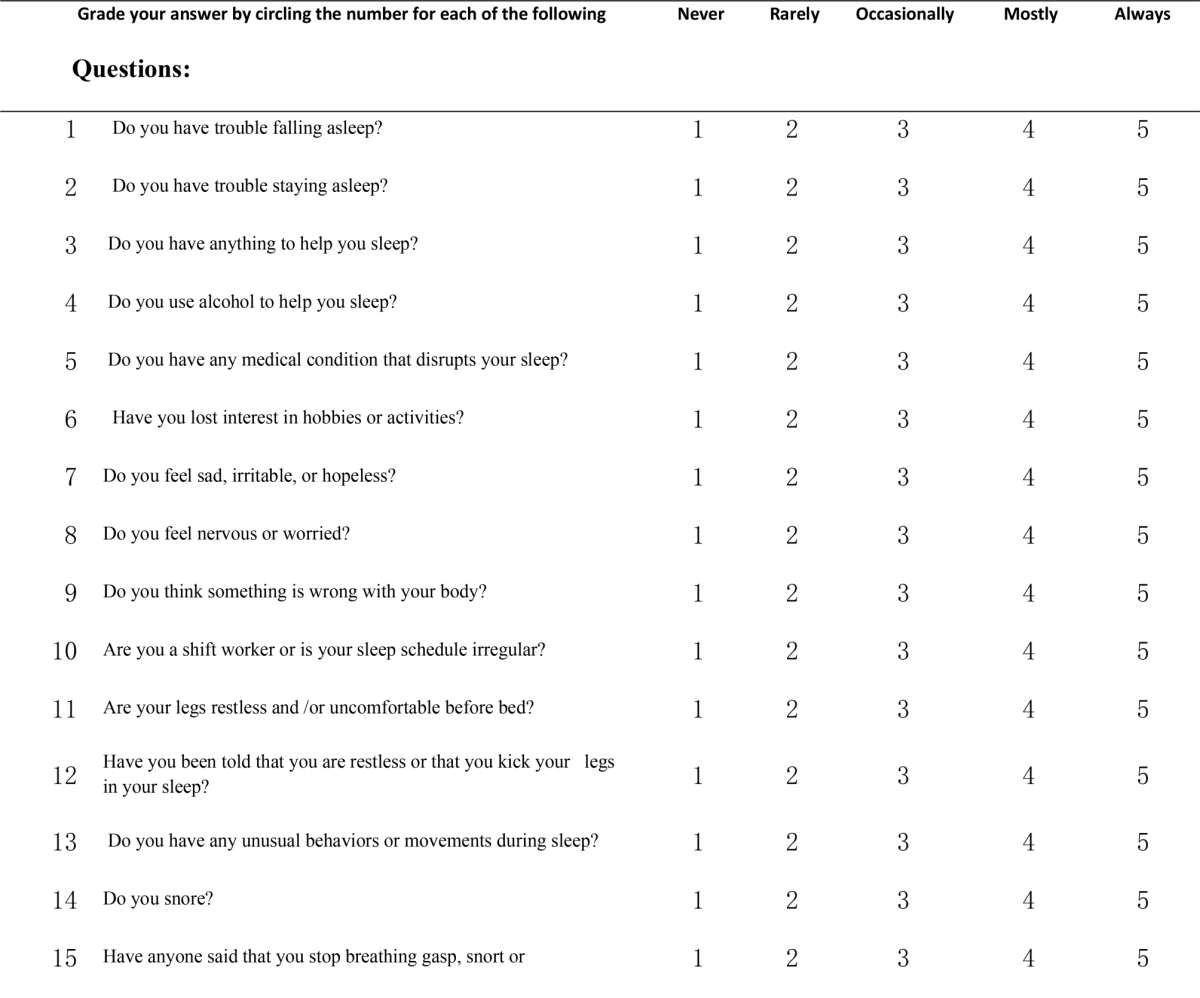
Sleep disorders questionaries

According to the International Classification of Sleeping Disorders (ICSD3), sleep disorders are classified mainly into parasomnias and insomnia which are further divided into sub types [31] sleep disorders classification. (Fig. 2). SLEEP DISORDERS QUESTIONNAIRE: This questionnaire is designed to assist doctors in screening for insomnia and identifying potential sleep disorders. However, a more comprehensive clinical assessment is required, and a referral to a specialist may be necessary. We have utilized questions 1–13, as shown in Fig. 3. The Diagnostic Domains are as follows: (1) Insomnia: Q1-5. (2) Psychiatric Disorders: Q6-9. (3) Circadian Rhythm Disorder: Q10. (4) Movement disorders: Q11-12. (5) Parasomnias: Q13. (detailed questionnaire in supplementary files).

### The zung self-rating anxiety scale

SAS is an assessment tool comprising of 20 questions aimed at evaluating anxiety levels in individuals [32]. The questions are categorized into four groups based on the experienced symptoms: cognitive, autonomic, motor, and central nervous system. It is a self-reporting test, which means that the individual completes it themselves. The scoring interpretations are as follows: 20–44 Normal Range, 45–59 Mild to Moderate Anxiety Levels, 60–74 Marked Severe Anxiety Levels, and 75–80 Extreme Anxiety Levels. (detailed questionnaire in supplementary files). Lindsay and Michie published a study in 1988 in the Journal of Mental Deficiency Research (32, 485–490), where they adopted the Zung Self-Rating Anxiety Scale (SAS) for individuals with intellectual disabilities (ID) [33]. Research has shown that individuals with intellectual disabilities (ID) often experience high levels of anxiety [34–36]. The SAS-ID can be useful for research and clinical purposes, such as assessing treatment effectiveness over time [37].

### The Beck depression inventory

(BDI, BDI-1 A, BI-II), created by Aaron T. Beck, is a 21-question choice self; one of the most widely used psychometric tests for measuring the severity of depression [38] 0.0–9: Indicates minimal depression,10–18: Indicates mild depression,19–29: Indicates moderate depression,30–63: Indicates severe. (detailed questionnaire in supplementary files). The Beck Depression Inventory (BDI) is a popular self-assessment tool to measure depression. It consists of 21 items and has been used in over 7,000 studies worldwide. The BDI was first proposed by Beck et al. and has undergone two major revisions: the BDI-IA in 1978 and the BDI-II in 1996 [39–43]. The BDI-II is a reliable and cost-effective psychometric tool that can distinguish between depressed and non-depressed individuals. It has improved validity, making it suitable for measuring depression severity in research and clinical settings worldwide [44].

### Statistical analysis

The sample size for this study was estimated using the following formula: n = Zα/2^2 x P (1-P)/d^2. Based on a previous studies [45–49], the prevalence of sleep disorders in patients with orthopedic trauma was estimated to be 40%. With a margin of error of 5%, a confidence level of 95%, and a non-response rate of 10%, the estimated sample size was 265.

A post-hoc power analysis was conducted to evaluate the statistical power of the study. The analysis showed that the study had a power of 80% to detect a significant difference in sleep disorders between different fracture types, assuming a significance level of 0.05.

In this study, several statistical analysis models and methods were used to examine the relationship between various factors and the outcomes of interest. The study employed ordinal logistic regression analysis to examine the relationship between various pelvic fractures and seven subscales of the Majeed Pelvic Score (MPS), as well as the Sleep Disorder Questionnaire (SDQ) and Beck Depression Inventory (BDI). The purpose of the ordinal logistic regression analysis was to determine whether there was a significant relationship between the independent variables (such as age, gender, cause of injury, post-operative complications, and injury severity) and the dependent variables (the MPS subscales, SDQ, and BDI).

Descriptive statistics were used to summarize the mean scores for each domain of the MPS questionnaire. The mean scores were calculated based on the responses of the patients to the questions in each domain of the MPS questionnaire.

Chi-square tests were used to determine whether there were significant differences in the prevalence of sleep disorders and psychiatric disorders among the different categories of pelvic fractures. The study used correlation analysis to examine the relationship between different factors, such as the correlation between the MPS scores and the SDQ and BDI scores.

In summary, the statistical analysis models and methods used in this study included ordinal logistic regression analysis, descriptive statistics, chi-square tests, and correlation analysis. These methods were used to examine the relationship between various factors and the outcomes of interest and to determine whether there were significant differences among the different categories of pelvic fractures. The results are presented as β-coefficients (B) with 95% CIs. All statistical analyses were performed using SPSS, version 22 (SPSS Inc, Chicago, IL, USA), with consultation from statistical experts. A p-value of 0.05 was considered statistically significant. Future studies with larger sample sizes and more robust statistical power are needed to further investigate the relationship between pelvic and acetabular fractures, sleep disorders, and psychological well-being.

## Results

### Demographic data

This table shows insights on patients with pelvic fractures and the associated risk factors. The study evaluated 216 patients with different types of pelvic fractures based on age, gender, ISS, and mechanism of injury. Most patients were male, with car accidents and falls from heights being the most common. MIPPO was the most common surgical approach used. The included patients’ average age was 48.24 years (SD 14.98), and the average ISS was 15.37 years (SD 8.07). Sixteen acetabulum fractures and 148 pelvic fractures (Tiles a, b, and c) were treated using the Mippo method. Four transverse acetabulum fractures were treated using the (MIPPO + Kocher-Langenbeck) approach, 28 posterior acetabulum wall fractures were treated using the KL approach, two pelvic Tile c-type fractures, seven pelvic and eight acetabulum fractures were treated using the ilioinguinal approach. 20 patients (9%) who arrived in the ER were hemodynamically unstable (shock class 3 or higher). Complex fracture patients had a markedly higher ISS and shock class and were more frequently operated on and were more frequently treated with operation/surgery). In 58% of patients, concurrent injuries were found. In 44 patients (32.4%), there were concurrent injuries to the lower extremities. 47 patients (34.5%) had neurological damage, of whom 28 (20.6%) had severe head trauma. Nine patients (6.6%) had focal neurological deficits. One Tile B patient had a urethral injury. The average follow-up time was two years, ranging from four to one. 39 patients diagnosed with deep vein thrombosis (DVT) due to pelvic fractures, and 8 patients with acetabulum fractures were treated according to hospital protocols for DVT, and all patients were stabilized. Two patients in Tile A, three in Tile B, and one in Tile C, as well as three patients with posterior wall fracture, reported experiencing numbness or irritation in their lower limbs. The Mippo Technique was used to treat Tiles A, B, and C, which involves a pelvic incision that can affect the skin and thigh. Posterior wall fracture and its surgical treatment can lead to sciatic nerve damage. Most of the symptoms went away with functional training or six months after surgery. However, one patient from Tile B reported experiencing some wound irritation one year after the surgery, but it had improved compared to the symptoms experienced six months after the surgery. (Patient characteristics are listed in (Table 1).

**Table 1:** Patients characteristics

Total number=216	Tile (pelvic fractures)			Letournel-Judget (acetabulum fractures)					
	Tile A	Tile B	Tile C	Ant wall	Ant column	Posterior wall	Transverse	Both columns	*p-value*
*N*	58	60	39	8	2	37	11	1	
Average Age	52.07	48.23	46.05	44.75	42.00	45.62	47.09	61.00	0.3993
Male (%)	38 [66]	37 [62]	28 (72)	4 [50]	2 (100)	31 (84)	6 [55]	1 (100)	
Female (%)	20 [34]	23 [38]	11 [28]	4 [50]	0	6 [16]	5 [45]	0	
ISS (*P*<0.001)	9.84	17	22	8.75	7.5	13.4	14.55	22	<0.001
**Mechanism of injury**									
Car accident (%)	30 [51]	31 [52]	10 [26]	2 [25]	1 [50]	13 [35]	7 [64]	0	
Fall from height (%)	16 [28]	15 [25]	21 [54]	3 [38]	1 [50]	19 [51]	3 [27]	1 (100)	
Fall from same level (%)	7 [12]	4 [7]	4 [11]	2 [25]	0	4 [11]	1 [9]	0	
Entrapment (%)	5 [9]	10 [17]	4 [11]	1 [13]	0	1 [3]	0	0	
**Surgical approaches**									
MIPPO (%)	53 (91)	59 (98)	36 (92)	5 [33]	1 [50]	3 [8]	8 (73)	0	
MIPPO+KL (%)	0	0	0	0	0	0	4 [36]	0	
Kocher-Langenbeck (%)	0	0	1 [3]	0	0	28 (76)	1 [9]	0	
Ilioinguinal (%)	4 [7]	1 [2]	2 [5]	0	1 [50]	6 [16]	1 [9]	0	
Para rectus (%)	1 [2]	0	0	3 [38]	0	0	0	1 (100)	
Neurological injury (%)	5 [9]	10 [17]	2 [5]			7 [19]			
Head trauma (%)	6 [10]	5 [8]	3 [8]			7 [19]			
Femoral neck fracture (%)	0	1 [2]	4 [10]		2 [50]	3 [8]	1 [9]	0	
Urogenital injury (%)	0	0	1 [3]						
Diabetic (%)	2 [3]	2 [3]	1 [3]			4 [11]	2 [18]		
CHD (%)	5 [9]	6 [10]	2 [5]			3 [8]			
DVT (%)	10 [17]	16 [27]	10 [26]	3 [38]		6 [16]	1 [9]	1 (100)	
Shock≥ Grade 3 (%)	0	4 [7]	8 [21]	2 [25]		6 [16]			
Limb Numbness/Irritation	2 [3]	3 [5]	1 [3]			3 [8]			

### Sleep disorders results

According to the sleep disorder questionnaires, neither somatization nor circadian rhythm disorder was noted in any pelvic or acetabulum fractures group. The rate of Insomnia was relatively higher in Tile B 24 (40%), and posterior acetabulum wall fractures 6(16%). Out of the 17 patients with pelvic fractures, those with Tile C had a higher incidence of sleep movement disorders. Similarly, among the 18 patients with acetabulum fractures, those with a posterior acetabular wall had a higher likelihood of experiencing sleep movement disorders Table 4.

### Depression and anxiety results

The Table 4 provides information on various sleep and mental health measures such as the Sleep Disorder Questionnaire (SDQ), Beck Depression Inventory (BDI), and Zung Self-Rating Anxiety Scale, along with some demographic information about the participants in each group such as gender and number of individuals. The results are presented differently depending on the measure being reported. For instance, the SDQ provides the percentages of participants with insomnia, psychiatric disorders, and movement disorders, while the BDI and Zung Self-Rating Anxiety Scale report mean scores and the percentage of individuals with different levels of depression or anxiety severity. The percentage of participants reporting insomnia and psychiatric disorders is relatively high across all groups, ranging from 16 to 40% and 5–10%, respectively. Some groups have higher mean scores on the BDI and Zung Self-Rating Anxiety Scale, indicating higher levels of depression or anxiety compared to other groups. The normal percentage of BDI and Zung Self-Rating Anxiety Scale scores is high for most of the groups, implying that many participants in each group are not experiencing significant levels of depression or anxiety. The contingency table reveals that the distributions of SDQ and Zung are different when there are different fracture groups, with respective p-values of (X2 = 29.255, p0.05), and (X2 = 25.958, p0.05). While there was no distinguishable correlation between anxiety and pelvic and acetabular fractures, Tile B and posterior acetabular wall fractures were more likely to have mild mood disturbance. Transverse acetabulum fracture (27%) and Tile A, B, and C (9%, 7%, and 10%), as well as the posterior wall (11%) all showed signs of borderline clinical depression.The study also investigated the relationship between various pelvic fractures and sleep disorders using the SDQ and BDI. The logistic regression was binary, with the outcome variable being whether or not the patient had a sleep disorder or depression. Table 4 shows the Sleep Disorder Questionnaire (SDQ), Beck Depression Inventory (BDI), and Zung Self-Rating Anxiety Scale.

**Table 2:** Majeed pelvic score (MPS) Questionnaire results

MPS scores of all patients were divided into different Tile (pelvic) and Letournel-Judget(acetabulum fractures) classification groups.										
MPS dimension	Description	MPS scoring	Tile A%	Tile B%	Tile C%	Anterior wall%	Anterior column%	Posterior wall%	Transverse%	Both columns%
**Pain**	Intense, continuous at rest	5								
	Intense with activity	10								
	Tolerable, but limits activity	15		1 [2]			1 [50]	1 [3]		
	Moderate activity, abolished by rest	20	18 [31]	6 [10]	4 [10]	1 [13]		3 [8]		
	Mild, intermittent, normal activity	25	13 [22]	7 [12]	4 [10]	1 [13]		9 [24]	3 [27]	1(100)
	Slight, occasional or no pain	30	27 [47]	46(77)	31(79)	6(75)	1 [50]	24 [65]	8(73)	
**Work**	No regular work	4	3 [4]	1 [2]	1 [3]		1 [50]			
	Light work	8	2 [3]	2 [3]	2 [5]			1 [3]		
	Change of job	12	2 [3]	2 [3]	2 [5]	1 [13]	1 [50]	1 [3]	1 [9]	
	Same job, reduced performance	16	28 [48]	29 [48]	10 [26]	3 [38]		17 [46]	5 [45]	1(100)
	Same job, same performance	20	23 [40]	19 [32]	24 [62]	4 [50]		18 [49]	5 [45]	
**Sitting**	Painful	4		1 [2]				2 [6]		
	Painful if prolonged or awkward	6	2 [3]	2 [3]		1 [13]				
	Uncomfortable	8	26 [45]	25 [41]	11 [28]	2 [25]	2(100)	10 [27]	3 [27]	1(100)
	Free	10	30 [52]	32 [53]	24 [62]	5 [63]		24 [65]	8(73)	
**Sexual intercourse**	Painful	1	4 [7]	2 [3]	2 [5]			3 [8]		
	Painful if prolonged or awkward	2	2 [3]	8 [13]	4 [10]	1 [13]	1 [50]	3 [8]	1 [9]	
	Uncomfortable	3	17 [29]	11 [18]	5 [13]		1 [50]	6 [16]	2 [18]	1(100)
	Free	4	35 [60]	38 [63]	26(67)	7(88)		25(68)	8(73)	
**Walking aids**	Bedridden or almost bedridden	2								
	Wheelchair	4								
	Two crutches	6								
	Two sticks	8			1 [3]					
	One stick	10	5 [9]	9 [15]	7 [18]	1 [12]		5 [14]	1 [9]	
	No sticks	12	53(91)	51(85)	31(79)	7(88)	2(100)	32(86)	10(91)	1(100)
**Gait unaided**	Cannot walk or can barely walk	2								
	Shuffling small steps	4			1 [3]		1 [50]			
	Gross limp	6	2 [3]		1 [3]			2 [5]		
	Moderate limp	8	6 [10]	5 [8]	6 [15]	1 [13]	1 [50]		1 [9]	
	Slight limp	10	14 [24]	34 [57]	16 [41]	4 [50]		15 [41]	6 [55]	1(100)
	Normal	12	36 [62]	21 [35]	15 [38]	3 [38]		20 [54]	4 [36]	
**Walking distance**	Bedridden or few meters	2								
	Very limited time and distance	4	1 [2]				1 [50]	1 [3]		
	Limited with sticks, difficult without prolonged standing possible	6	2 [3]	1 [2]	2 [5]					
	One hour with a stick, limited without	8	4 [7]	4 [7]	1 [3]			1 [3]		
	One hour without sticks, slight pain or limp	10	34 [59]	34 [57]	24 [62]	4 [50]	1 [50]	23 [62]	6 [55]	1(100)
	Normal for age and general condition	12	11 [19]	21 [35]	12 [31]	4 [50]		12 [32]	5 [45]	
**Majeed pelvic score rank**	51-60		1 [2]		2 [5]		1 [50]	1 [3]		
	61-70		2 [3]	2 [3]	1 [3]					
	71-80		4 [7]	9 [15]	3 [8]		1 [50]	3 [8]		
	81-90		21 [36]	13 [22]	9 [23]	3 [38]		10 [27]	4 [36]	1(100)
	91-100		30 [52]	36 [60]	24 [62]	5 [63]		23 [62]	7 [64]	

After walking for an hour, most patients experienced only mild pain or limping. Following surgery, Tile A, Tile B, and Tile C patients were able to return to work. The average MPS score was 89.68±10.04. MPS scores range from 51 to 100, with rankings based on groups of ten. Of the total patients, 186 (86%) scored higher than 80

### MPS functional outcome scores

The table presents the results of an Ordinal logistic regression analysis of Majeed pelvic score (MPS) with various factors such as pain, work, sitting, sexual intercourse, standing, gait unaided, walking distance, age, sex, ISS, and fracture type. The table is divided into two sections with the first section presenting the regression coefficients, 95% confidence intervals, and p-values for pain, work, sitting, and sexual intercourse for each of the factors. The second section presents the regression coefficients, 95% confidence intervals, and p-values for standing, gait unaided, walking distance, and MPS score for each of the factors. The fracture type is further divided into four categories, Tile A, Tile B, Tile C, and Acetabular, and the table provides the regression coefficients, 95% confidence intervals, and p-values for each fracture type. The table compares the various fracture types and their impact on pain, work, sitting, sexual intercourse, standing, gait unaided, walking distance, and MPS score. The study found that fractures classified as Tile A, B, and C are associated with lower mobility issues, self-care problems, pain and discomfort scores, and fewer problems with usual activity when compared to acetabular fractures. Specifically, Tile B fractures were found to have a significantly lower rate of usual activity issues compared to acetabular fractures. Additionally, after surgery, a high percentage of patients with each type of fracture were able to return to work − 88% for Tile A, 80% for Tile B, and 86% for Tile C. Overall, the study suggests that Tile A, B, and C fractures may have better outcomes in terms of mobility, self-care, and pain compared to acetabular fractures. The beta coefficients for mobility, self-care, usual activity, and pain and discomfort were − 1.448 (95%CI: -2.221-0.674), -1.259 (95%CI: -2.191-0.326), -1.020 (95%CI: -1.795-0.244), and − 1.037 (95% CI: -1.771-0.303), respectively, for Tile A fractures. The corresponding beta coefficients for Tile B fractures were − 2.545 (95%CI: -3.511-1.579), -1.828 (95%CI: -2.865-0.792), -1.020 (95%CI: -1.795-0.244), and − 1.641 (95% CI: -2.402-0.879), respectively. For Tile C fractures, the beta coefficients were − 1.997 (95%CI: -3.049-0.945), -1.496 (95%CI: -2.648-0.343), and − 1.332 (95% CI: -2.243-0.420) for mobility, self-care, and pain and discomfort, respectively. Each of the 216 patients finished the MPS, and the median score was 89.68 ± 10.04. After an hour of walking, more than 50% of patients with every fracture type displayed a slight limp. However, following surgery, the majority of patients were able to return to their jobs, with 88% of Tile A, 80% of Tile B, and 86% of Tile C patients successfully returning to their jobs. The confidence intervals provide a range of plausible values for the true effect size or beta coefficient, which can help us assess the level of uncertainty in the results.

According to our findings, a positive relationship exists between age and the degree to which pain, work, sexual activity, and walking distance are affected. At the same time, there is a negative relationship between age and the total MPS score. The average MPS scores did not significantly differ between the various types of fractures. dummy variables were used in the logistic regression analysis to represent the different types of pelvic fractures. (Majeed Pelvic Score (MPS) questionnaire results and employed ordinal logistic regression of MPS. Tables (2 and 4).

**Table 3:** Ordinal logistic regression of MPS

	Pain			Work			Sitting			Sexual Intercourse		
	β-coefficient	95%CI	p-value	β-coefficient	95%CI	p-value	β-coefficient	95%CI	p-value	β-coefficient	95%CI	p-value
**Age**	0.021	0.001~0.041	0.040	0.022	0.004~0.039	0.016	0.006	-0.012~0.025	0.484	0.021	0.002~0.040	0.033
**Sex(Female)**	0.438	-0.164~1.041	0.154	0.014	-0.539~0.567	0.961	0.262	-0.313~0.837	0.372	-0.147	-0.751~0.458	0.634
**ISS**	0.001	-0.043~0.046	0.950	0.019	-0.021~0.059	0.350	0.027	-0.014~0.068	0.202	0.005	-0.039~0.048	0.825
**Fracture type**												
**Tile A**	-1.186	-1.958~-0.415	0.003	0.260	-0.442~0.961	0.468	-0.475	-1.209~0.260	0.205	0.231	-0.491~0.954	0.530
**Tile B**	-1.487	-2.295~-0.680	<0.001	-0.651	-1.378~-0.076	0.079	-0.812	-1.567~-0.056	0.035	-0.874	-1.685~-0.063	0.035
**Tile C**	-1.136	-2.096~-0.175	0.021	0.042	-0.829~0.913	0.925	-0.673	-1.583~0.236	0.147	-0.441	-1.389~0.507	0.362
**Acetabular**	0*			0*			0*			0*		
	**Standing**			**Gait Unaided**			**Walking Distance**			**Majeed Pelvic Score rank**		
	β-coefficient	95%CI	p-value	β-coefficient	95%CI	p-value	β-coefficient	95%CI	p-value	β-coefficient	95%CI	p-value
**Age**	0.020	-0.009~0.049	0.176	0.005	-0.012~0.022	0.584	0.027	0.008~0.045	0.005	-0.035	-0.051~-0.019	<0.001
**Sex(Female)**	0.507	-0.325~1.339	0.232	-0.054	-0.607~0.500	0.849	0.205	-0.375~0.785	0.811	0.005	-0.031~0.041	0.779
**ISS**	0.023	-0.041~0.087	0.482	0.027	-0.013~0.067	0.179	0.005	-0.036~0.047	0.488	-0.306	-0.810~0.197	0.233
**Fracture type**												
**Tile A**	1.366	0.202~2.529	0.210	0.686	-0.028~1.400	0.060	-0.897	-1.657~-0.137	0.021	-0.509	-1.154~0.136	0.122
**Tile B**	0.076	-1.221~1.373	0.909	0.098	-0.625~0.821	0.790	-1.232	-2.010~-0.455	0.002	-0.208	-0.859~0.443	0.531
**Tile C**	0.282	-1.177~1.742	0.705	0.335	-0.540~1.211	0.453	-1.014	-1.954~-0.075	0.034	0.328	-0.474~1.130	0.423
**Acetabular**	0*			0*			0*			0*		

Tile A B C fractures are less painful and allow for greater walking distance compared to Acetabular fractures. Tile B fractures have less likelihood of issues with work, sitting, and sexual intercourse. The values obtained for β=-0.651 (95%CI: -1.378~0.076), β=-0.812 (95%CI: -1.567~-0.056), and β=-0.874 (95%CI: -1.685~-0.063) respectively. More aged patients were located in lower MPS rank with a value of β=-0.035 (95%CI: -0.051~-0.019). The scoring was done into an ordered variable (Table 2) with 5 ranks: 51-60, 61-70, 71-80, 81-90, and 91-100. Then, ordinal logistic regression was performed

**Table 4:** Sleep Disorder Questionnaire (SDQ), Beck Depression Inventory (BDI), Zung Self-Rating Anxiety scale score and p-value

	Tile A	Tile B	Tile C	Ant wall	Ant column	Posterior wall	Transverse	Both columns	X²	p-value
*N*	58	60	39	8	2	37	11	1		
SDQ									29.255	0.01
Insomnia (%)	19 [33]	24 [40]	10 [26]	2 [25]	0	6 [16]	1 [9]	0		
Psychiatric disorder (%)	3 [5]	5 [8]	4 [10]	0	0	4 [10]	2 [18]	0		
Circadian Rhythm (%)	0	0	0	0	0	0	0	0		
Movement disorders (%)	5 [9]	3 [5]	9 [23]	2 [25]	1 [50]	12 [32]	2 [18]			
Somatization (%)	0	0	0	0	0	0	0	0		
BDI(SD)	9.22±3.99	11.65±4.69	11.90±4.14	9.13±3.13	14±0.00	12.38±4.16	11.91±5.59	15±0.00	19.309	0.153
Normal (%)	32 [55]	29 [48]	16 [41]	0	0	13 [35]	5 [46]	0		
Mild Mood Disturbance (%)	21 [36]	27 [45]	19 [49]	4 [50]	2(100)	20 [54]	3 [27]	1 [1]		
Borderline Clinical depression (%)	5 [9]	4 [7]	4 [10]	0	0	4 [11]	3 [27]	0		
Moderate depression (%)	0	0	0	0	0	0	0	0		
Severe Depression (%)	0	0	0	0	0	0	0	0		
Extreme Depression (%)	0	0	0	0	0	0	0	0		
Zung(SD)	30.71±17.55	30.93±19.42	22.38±16.50	22.38±16.50	3.00±1.41	46.65±17.80	41.36±25.43	56.00±0.00	25.958	0.026
Normal (%)	44(76)	40(67)	18 [46]	8(100)	2(100)	20 [54]	6 [55]	0		
Minimal to Moderate (%)	13 [22]	19 [31]	18 [46]	1 [13]	0	17 [46]	3 [27]	1(100)		
Severe Anxiety (%)	1 [2]	1 [2]	3 [8]	0	0	0	2 [18]	0		
Most extreme (%)	0	0	0	0	0	0	0	0		

## Discussion

Our study found that many patients with Tile b c and posterior acetabulum wall fractures experienced mild mood disturbances. Compare to study of Martin MP et al. higher levels of depression and anxiety symptoms were associated with poorer functional outcomes in patients with Tile C pelvic injuries [50] but we didn’t find though no severe depression, anxiety or somatization disorders were observed, some types of fractures may be associated with borderline clinical depression. In a diverse cohort of orthopedic trauma patients, clinically relevant depression was prevalent at a rate of close to 45%. Depression and overall disability have a strong relationship. The risk of depression may also rise in the presence of an open fracture [51]. Chronic pelvic pain is unknowingly linked to sleep issues, depression, and anxiety [52].In our study, we found that Insomnia was more common in Tile B 24, affecting 40% of the patients. We also observed that sleep movement disorders were more frequent in patients with Tile C pelvic fractures. Similarly, patients with posterior acetabular wall fractures were more likely to experience sleep movement disorders. Our study results were similar to LU K et al’s regarding sleeping disturbance, but with gender specificity and time difference in their study results. Our study’s results were taken one year postoperative, and more than half of all patients still reported having trouble sleeping. LU K’s study, on the other hand, found that sleep disturbances were more likely to affect women than men, but their results were taken three months after surgery [49]. Women may be more susceptible to insomnia after trauma, with a strong association found among women but not men (Nicole A. et al.) [53]. In a study by Matthew C Swann et al. their findings suggest that sleep disturbance is both highly prevalent in Pittsburgh sleep quality index (86%) and severe (54.6% ) in patients recovering from a traumatic orthopedic injury [47]. Stephen Breazeale et al. discovered four symptom cluster profiles that they categorized as Physical Symptoms Only, Mild, Moderate, and Severe Psychological Distress in orthopedic trauma patients. Pelvic injuries can cause long-lasting physical pain and mental health issues. Participants in a study conducted by Kenleigh R reported higher levels of PTSD, depression, and problematic alcohol use one year after injury [54]. A study by Zhen Hong et al. found that 28.20% of 468 patients with traumatic fractures had acute stress disorder (ASD) [55].

Another study by Shalev et al. (2017) explored the relationship between comorbid PTSD and depression on psychological well-being and functional outcomes following orthopedic trauma. The study found that patients with comorbid PTSD and depression experienced more symptoms, felt more distress from those symptoms, and performed worse in daily life than patients with only one of these conditions. While our study did not find a significant correlation between anxiety and pelvic and acetabular fractures, the study by Shalev et al. highlights the importance of exploring comorbid conditions in orthopedic trauma patients [56]. When compared to acetabular fracture, Tile B fractures are less likely to cause problems with work, sitting, and sexual activity According to our findings, there is a positive association between age and the degree to which a person experiences pain, works, has sex, and walks a distance, whereas there is a negative correlation between age and the total MPS score. Studies have shown that age-related changes in the body, such as hormonal changes and decreased muscle mass, can lead to these issues [57]. Chronic pain is a common health problem in older adults, with prevalence rates ranging from 25–76% [58]. As people age, their body tissues may become less resilient, leading to increased risk of injury and chronic pain. Chronic pain can limit physical activity and impair mobility, making it difficult for older adults to engage in work or leisure activities [59].Reduced mobility and physical activity are also common in older adults, with studies showing that physical activity levels decline with age [60]. Sexual dysfunction is another issue that becomes more common as people age, with studies indicating that up to 40% of older adults experience sexual problems [61]. Patients with pelvic ring injuries have reasonable long-term physical functioning and quality of life, but it is significantly lower compared to other groups in the general population [62]. Screening injured patients and providing timely intervention for posttraumatic stress disorder (PTSD) and depression could improve outcomes and quality of life [63].Our study revealed that patients with pelvic fractures had better results in the MPS scores compared to those with acetabular fractures. The pelvic fracture group demonstrated superior performance in walking, distances, work, sitting, and sexual activities compared to the acetabular fracture group. These findings suggest that pelvic fractures may have a better prognosis and improved functional outcomes compared to acetabular fractures.As per our earlier results, patients who had acetabular fractures recorded lower scores on the Majeed Pelvic Score (MPS) in comparison to patients with other types of pelvic fractures. The complex anatomy of the acetabulum and the difficulty involved in surgical repair may contribute to worse outcomes. Additionally, acetabular fractures are often caused by high-energy trauma and can be associated with other injuries or complications [64]. Some people may have negative reactions to surgical implants, such as allergies to metals, methacrylate’s, and antibiotics those with a history of material reactions should undergo pre-implant testing to explore alternative options [65]. In our study some individuals reported numbness and irritation in the implant area and their thighs. Identification of the root cause and appropriate treatment to alleviate any numbness or irritation is crucial. A study by Katherine F et al. found that sexual function was notably reduced after experiencing a pelvic fracture, with a significant decrease in the quality of life. Sexual dysfunction is an independent risk factor for decreased quality of life following the injury [66]. However, many of the patients included in our study did not answer questions related to sexual activities, which is considered an essential factor for of life as included in our questionnaires.

### Limitations

Due to the small number of patients with acetabulum subgroup fractures and the fact that most patients’ information was either missing or inaccurate, we did not perform a comparative study of various approaches to treating pelvic and acetabulum fractures. The questionnaires did not address the socioeconomic problems that have a significant impact on people’s psychological well-being. The concurrent injuries were present in 58% of the patients, which could act as a major confounding factor and may have influenced the results and conclusions of the study. Further comparative studies are needed to confirm the psychological and health-related issues and reduction quality in pelvic and acetabulum fractures treated with different approaches. Most middle-aged patients and patients over 60 or 70 years old didn’t respond to sex questions due to the culture and privacy.

### Conclusion

Our study found that 80% of patients showed better mobility and comfort in performing daily activities after surgical intervention for pelvic and acetabular fractures. However, older and middle-aged patients may experience anxiety and depression. Also, certain types of fractures were associated with an increased risk for insomnia and sleep movement disorders. Pelvic and acetabular fracture patients may experience borderline clinical depression. Anxiety levels do not seem to be significantly associated with these fractures. Understanding these psychological challenges can aid medical professionals in creating personalized treatment plans. Our study highlights the importance of a multidisciplinary approach to care for orthopedic trauma patients with pelvic injuries. Psychological screening and intervention should be integrated into their recovery. It is especially important to monitor patients with posterior acetabulum wall fractures and Tile-C pelvic fractures.

### Electronic supplementary material

Below is the link to the electronic supplementary material.

Supplementary Material 1

Supplementary Material 2

Supplementary Material 3

## Data Availability

Researchers who make a valid request to the corresponding author will be given access to the data.
