# Supplementary material for: Sleep quality and psychological health in patients with pelvic and acetabulum fractures: a cross-sectional study
Source: BMC Geriatr. 2024 Apr 4;24:314. doi: 10.1186/s12877-024-04929-y (PMC10993547; doi:10.1186/s12877-024-04929-y)
Supplement: Supplementary file 2 — Supplementary Material 2 [file 12877_2024_4929_MOESM2_ESM.pdf]

NAME ____________________ DATE ____________________

Zung Anxiety Self-Assessment Scale

	None or a little of the time	Some of the time	Good part of the time	Most or all of the time
1. I feel more nervous and anxious than usual	1	2	3	4
2. I feel afraid for no reason at all	1	2	3	4
3. I get upset easily or feel panicky	1	2	3	4
4. I feel like I'm falling apart and going to pieces	1	2	3	4
5. I feel that everything is all right and nothing bad will happen	4	3	2	1
6. My arms and legs shake and tremble	1	2	3	4
7. I am bothered by headaches, neck and back pains	1	2	3	4
8. I feel weak and get tired easily	1	2	3	4
9. I feel calm and and can sit still easily	4	3	2	1
10. I can feel my heart beating fast	1	2	3	4
11. I am bothered by dizzy spells	1	2	3	4
12. I have fainting spells or feel faint	1	2	3	4
13. I can breath in and out easily	4	3	2	1
14. I get feelings of numbness and tingling in my fingers and toes	1	2	3	4
15. I am bothered by stomachaches or indigestion	1	2	3	4
16. I have to empty my bladder often	1	2	3	4
17. My hands are usually dry and warm	4	3	2	1
18. My face gets hot and blushes	1	2	3	4
19. I fall asleep easily and get a good night's rest	4	3	2	1
20. I have nightmares	1	2	3	4

Converting Raw Score Total to Anxiety Index

RAW SCORE	ANXIETY INDEX	RAW SCORE	ANXIETY INDEX	RAW SCORE	ANXIETY INDEX
20	25	40	50	60	75
21	26	41	51	61	76
22	28	42	53	62	78
23	29	43	54	63	79
24	30	44	55	64	80
25	31	45	56	65	81
26	33	46	58	66	83
27	34	47	59	67	84
28	35	48	60	68	85
29	36	49	61	69	86
30	38	50	63	70	88
31	39	51	64	71	89
32	40	52	65	72	90
33	41	53	66	73	91
34	43	54	68	74	92
35	44	55	69	75	94
36	45	56	70	76	95
37	46	57	71	77	96
38	48	58	73	78	98
39	49	59	74	79	99
				80	100

Raw Score Total [ ] Anxiety Index [ ]

Interpreting the Anxiety Index

Anxiety Index	Clinical Interpretation
Below 45	Within normal range
45 – 59	Minimal to moderate anxiety
60 – 74	Marked to severe anxiety
75 and over	Most extreme anxiety

- Check that all statements have been answered
- Scoring values are printed next to the response
- Add up the Raw Total Score
- Convert the Raw Total to the Anxiety Index

Instruction for use: (Zung Anxiety Assessment Tool)

1. The same caregiver should administer this test each time.
2. Choose a quiet place, preferably the same location each time the test is administered.
3. The administration of this test should not be immediately after some mental trauma or unsteady period.
4. Speak in a soft, pleasant tone.
5. Answer all questions by placing a check in the box to the left of the number under the appropriate answer.
6. Add the Raw Score values (numbers to the right of the check) for all questions and record the total in the "RAW SCORE TOTAL" box.
7. Compare the raw score to the anxiety index on the conversion chart and record the corresponding anxiety index in the "ANXIETY INDEX" box.
8. Compare the anxiety index with the clinical interpretation chart.
